# Altered Caffeine Metabolism Is Associated With Recurrent Hypoglycemia in Type 2 Diabetes Mellitus: A UPLC–MS-Based Untargeted Metabolomics Study

**DOI:** 10.3389/fendo.2022.843556

**Published:** 2022-06-17

**Authors:** Wang Lijing, Ke Sujie, Wang Linxi, Huang Lishan, Qi Liqin, Zhan Zhidong, Wu Kejun, Zhang Mengjun, Liu Xiaoying, Liu Xiaohong, Liu Libin

**Affiliations:** ^1^ Department of Endocrinology, Fujian Medical University Union Hospital, Fuzhou, China; ^2^ The School of Pharmacy, Fujian Medical University, Fuzhou, China

**Keywords:** Type 2 diabetes mellitus, recurrent hypoglycemia, metabolomics, liquid chromatography-mass spectrometry, caffeine

## Abstract

**Background:**

Recurrent hypoglycemia (RH) is well known to impair awareness of hypoglycemia and increase the risk of severe hypoglycemia; the underlying mechanism requires further understanding. We aimed to investigate the metabolic characteristic profile for RH in type 2 diabetes mellitus (T2DM) patients and explore the potential metabolic mechanism and prevention strategies.

**Methods:**

We screened 553 community-based T2DM patients. T2DM with RH (DH group, *n*=40) and T2DM without hypoglycemia (DC group, *n*=40) were assigned in the case-control study, matched by propensity score matching. Non-targeted, global metabolite profiling was conducted using ultra-high performance liquid chromatography-mass spectrometry. Principal component analysis and supervised projections to latent structures-discriminant analysis were constructed to evaluate the potential biomarkers. Metabolites with a fold change of >2.0 or <0.5, a t-test *q*-value <0.05, and variable importance in projection value of >1 were identified as significantly differential metabolites. MetaboAnalyst was performed to analyze the related metabolic pathways.

**Results:**

We identified 12 significantly distinct metabolites as potential biomarkers of RH, which were enriched in five pathways; the caffeine metabolic pathway was the most dominant related one. Caffeine and its main downstream metabolites (theophylline and paraxanthine, all *q <*0.05) were significantly lower during RH. The combination of these metabolites can serve as a reliable predictor biomarker for RH (area under the curve = 0.88). Regarding lipid metabolism, triglyceride was upregulated (*P*=0.003) and the O-Acylcarnitine was downregulated (*q* < 0.001). Besides, RH was accompanied by lower phenylalanine (*q*=0.003) and higher cortisone (*q*=0.005) levels.

**Conclusions:**

RH in T2DM is accompanied by caffeine, lipolysis, phenylalanine, and cortisone metabolism abnormalities. Caffeine might be a reliable candidate biomarker and potential prevention strategy for RH, but further validation studies are needed.

**Clinical Trial Registry:**

Chi CTR 1900026361, 2019-10-3.

## Introduction

Iatrogenic hypoglycemia is a major obstacle in the glycemic management of diabetes (1–3). Hypoglycemia episodes in type 2 diabetes mellitus (T2DM) arouse increasing attention because the risk is more treatment-dependent and less inevitable (4). Acute hypoglycemia trigger rapid counter-regulatory responses, including catecholamine response, sympathetic nerve stimulation, and hormone secretion, resulting in limited glucose utilization and the activation of gluconeogenesis to maintain homeostasis (5–7).The cumulative impact of recurrent hypoglycemia (RH) is focused on impaired awareness of hypoglycemia (IAH). IAH is characterized by lower glycemia thresholds of counter-regulatory responses (8), markedly increasing the risk of severe hypoglycemia up to six-fold (9). Thus far, the clinical approaches to IAH treatment are limited to patient education, frequent glucose monitoring, and appropriate adjustment of the therapy target (10). The underlying mechanism remains unclear, but includes alternatives of fuel transport and/or storage (11, 12), neuronal modulators (13, 14), changes in the transduction of the energy signal into neuronal firing rates (15), may involve multiple steps in the integrative networks that control glucose homeostasis (8). RH may lead to energy adaptations that allow lactate or ketones to be used as alternative fuels to maintain brain function (11, 16), thereby impairing hypoglycemia sensing simultaneously (17). Many of these findings are derived from animal research; however, new clues may emerge from research regarding the process of metabolic perturbations of RH in T2DM patients.

Metabolomics detects the overall and dynamic changes in endogenous small-molecule metabolites and provides an excellent approach to understanding the pathophysiology of the disease. During the last decade, ultra-high performance liquid chromatography-mass spectrometry (UPLC–MS) has been widely used for exploring potential biomarkers for the predictors and novel therapeutic targets in metabolic diseases such as DM (18–20). However, to the best of our knowledge, only two studies have investigated the metabolic changes associated with hypoglycemia in humans. Halama et al. were the first to report a comprehensive description of metabolomic changes within 24 hours of insulin-induced hypoglycemia in T2DM patients (21). McCarthy et al. explored the metabolomic responses to acute hypoglycemia during exercise in T1DM with greater adenosine salvage pathway activity and an increased utilization of glucogenic amino acids (22). These studies presented a metabolomics profile with acute artificial hypoglycemia in laboratory conditions, but they did not address the long-term or cumulative impact of hypoglycemia on the metabolomic profile and whether similar results can be achieved in real-world settings. In the present study, we screened a total of 553 community-based T2DM patients and conducted a case-control study to explore the serum characteristic metabolite signatures associated with RH using UPLC-MS. This investigation will help explore the underlying molecular mechanisms of RH in T2DM and provide new strategies for the management of RH.

## Methods

### Study Population

A total of 553 patients aged 35–75 years with T2DM (diagnosed using the 1999 World Health Organization criteria) who were undergoing follow-up at six community health service centers in November 2019 in Fujian Province, China, were screened. The exclusion criteria were as follows: patients with T1DM, gestational diabetes, acute complications of diabetes, malignant tumors, and poor compliance. All patients signed informed consent forms. The trial was approved by the Ethics Committee of Fujian Medical University Union Hospital and registered at the Chinese Clinical Trial Registry (Chi CTR 1900026361, date of registration: 2019-10-3).

**Figure 1:**
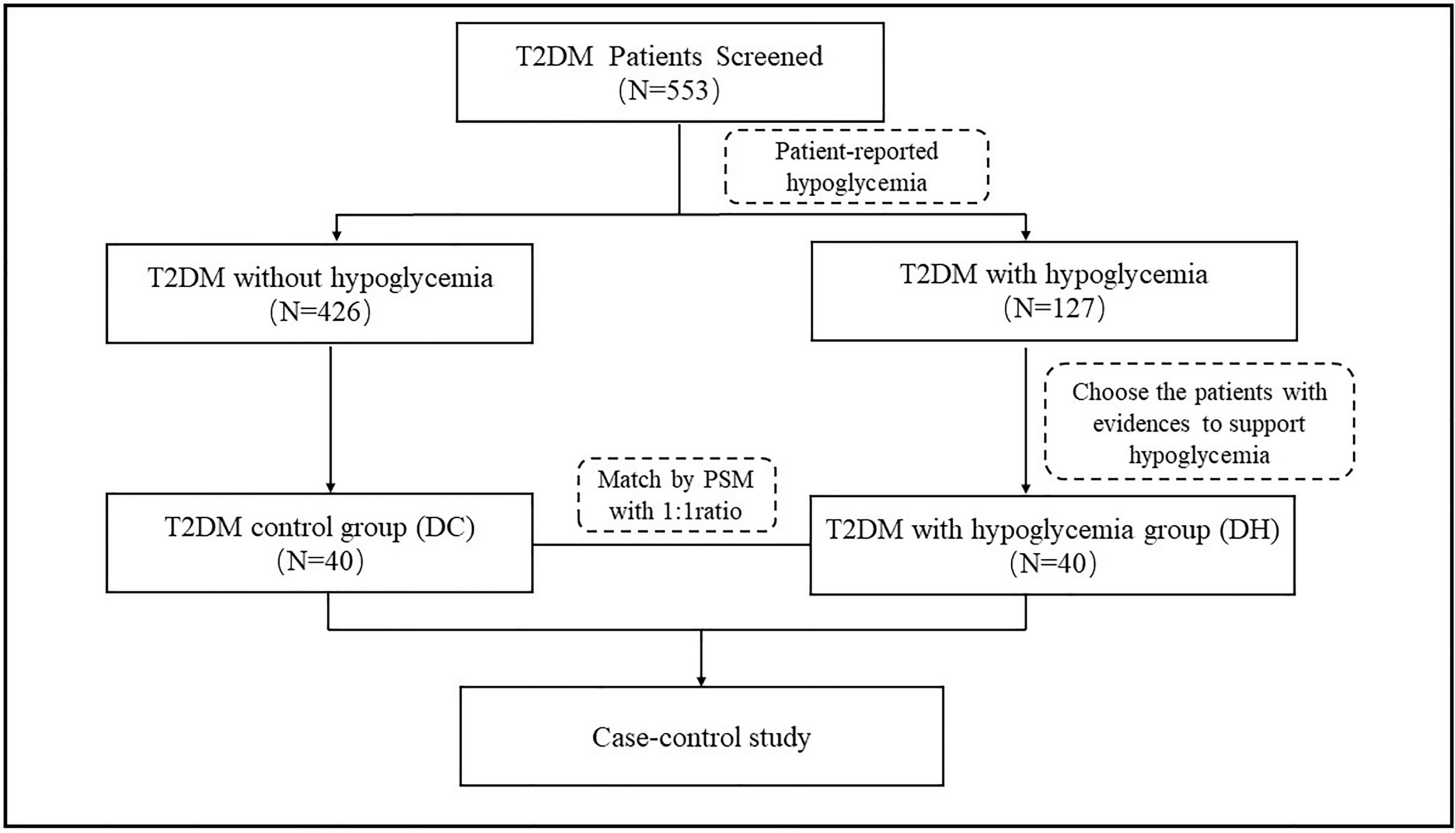
Selection of study participants.

There were 127 patients with self-reported hypoglycemia events within 6 months prior to this study; each of these patients completed a questionnaire about the details of their hypoglycemic episodes by recall. Based on the questionnaires responses, we identified 40 patients who had experienced at least two hypoglycemia events (diagnosed according to the American Diabetes Association criteria of hypoglycemia (23) with a blood glucose concentration <70mg/dL [3.9 mmol/L] or a severe hypoglycemia event characterized by altered mental and/or physical functioning that required assistance from another person for recovery) and placed them in the T2DM patients with hypoglycemia (DH) group. Propensity score matching (PSM) was performed at a 1:1 ratio to select 40 participants among the T2DM patients without any reported hypoglycemia episodes; this group is referred to as the T2DM control (DC) group. We adjusted for the following potential confounders: sex, age, duration of diabetes, blood pressure, body mass index (BMI), and treatment with glucose-lowering agents. The case screening process is illustrated in **Figure 1**.

### Data Collection

Demographic data collected included age, sex, education level, lifestyle factors, BMI, blood pressure, duration of T2DM and hypertension, and details of diabetes treatment. The consumption of tea or coffee during the previous 6 months was recorded in three categories: less than once a week (occasionally), one to six times a week (often), and more than once a day (always). Fasting blood samples were obtained and centrifuged at 3000 g for 10 minutes (4°C) to separate the serum from whole blood. Serum was stored at -80°C. The levels of fasting blood glucose, fasting C-peptide, total cholesterol (TC), triglycerides, low-density lipoprotein cholesterol (LDL-c), high-density lipoprotein cholesterol (HDL-c), and uric acid were measured using an automatic biochemical analyzer (Beckman AU5821, USA). Glycated hemoglobin (HbA1c) levels were determined using an automatic hemoglobin analyzer. The estimated glomerular filtration rate (eGFR) was calculated using the Chronic Kidney Disease Epidemiology Collaboration (CKD-EPI) formula (24).

### Metabolite Measurements Using UPLC-MS

The collected samples were thawed and dissolved at 4°C. We then added 50% methanol to precipitate the proteins on ice. Quality control (QC) samples were prepared using the same procedure as the test samples. All chromatographic separations were performed using a UPLC system (SCIEX, UK). An ACQUITY UPLC T3 column (100 × 2.1 mm, 1.8 μm; Waters, UK) was used for reverse-phase separation. The mobile phase consisted of solvent A (water, 0.1% formic acid) and solvent B (Acetonitrile, 0.1% formic acid). The flow rate was 0.4 mL/min. The column oven was maintained at 35°C. A high-resolution tandem mass spectrometer Triple TOF5600plus (SCIEX, UK) was used to detect metabolites eluted from the column. The Q-TOF was conducted in both positive and negative ion modes. The curtain gas was set at 30 psi. Ion source gas 1 was set at 60 psi, and ion source gas 2 was set at 60 psi. For the positive ion mode, the ion spray voltage floating field was set at 5000 V, while that for the negative ion mode was set at -4500 V. Mass spectrometry data were acquired. The total cycle time was fixed at 0.56 s. Four bins were summed for each scan at a pulser frequency value of 11 kHz by monitoring the 40 GHz multichannel time-to-digital converter detector with four-anode/channel detection. The mass accuracy was calibrated every 20 samples during the acquisition. A QC sample was acquired after every 10 samples (Pool of all samples) to evaluate the stability of the LC‐MS during the whole acquisition. All samples including QC were tested in the same batch.

### Metabolites Identification

The acquired MS data preprocessing including peak grouping, peak picking, second peak grouping, retention time correction and annotation of isotopes. LC−MS raw data files were converted into mzXML format and then processed by XC mass spectrometry (XCMS) software (www.bioconductor.org/), CAMERA and metaX toolbox implemented with the R software. We generated a peak table by combining the retention time, mass to charge (m/z) data, and corresponding peak intensity as the relative content of the metabolites. By matching the exact molecular mass data (m/z) of samples with that on the database, the Kyoto Encyclopedia of Genes and Genomes (KEGG) database (https://www.genome.jp/kegg/) and Human Metabolome Database (HMDB) (http://www.hmdb.ca/) were used to annotate the metabolites. Then we used the in-house secondary mass spectrometry library to match with the MS secondary fragmentation data to obtain more reliable identification results. Those features that were detected in less than 80% of biological samples or 50% of QC samples were removed. To further improve the data quality, the k‐nearest neighbor algorithm was used to supplement the remaining peaks with missing values. and then corrected by PQN (Probabilistic Classification) and QC-RSC (QC-robust spline Batch Correction).LC/MS and untargeted metabolomics raw data were performed at LC-Bio Technology Co., Ltd, Hangzhou, Zhejiang Province, China.

### Statistical Analyses

Statistical analyses was performed using the Software Package for Social Sciences software (SPSS) (version 24.0; IBM Inc., Armonk, NY, USA). Continuous data are reported as mean ± standard deviation, while categorical data are presented as numbers (n) and percentages (%) of the total data. To control for selection bias in the case-control study of metabolomic changes, we conducted 1:1 PSM using the nearest neighbor method, with a caliper of 0.08. Continuous variables were compared using the independent-samples t-test in the unmatched cohort, while the paired *t*-test or the Wilcoxon signed-rank test was used in the matched cohort. The chi-square test was performed for categorical variables in the unpaired cohort, while McNemar’s and Wilcoxon signed rank test were performed for categorical variables in the matched cohort. All statistical tests were two-sided and regarded as exploratory, with the criterion for statistical significance set at *P <*0.05.

Student’s t-tests were conducted to detect differences in metabolite relative contents between DC and DH groups, and the *P*-value was adjusted for multiple tests using a false discovery rate (the Benjamini-Hochberg Procedure) to get q-value, and q-value < 0.05 was considered as significant differences (25).Multivariate analysis including unsupervised principal component analysis (PCA) and supervised projections to latent structures-discriminant analysis (PLS-DA). PCA was constructed to demonstrate the distribution of origin data, while PLS-DA model was used to maximize class separation and determine which metabolites best distinguished between groups. The stability of the model was evaluated using cross-validation. The variable importance in the project (VIP) was obtained by the PLS-DA model. A fold change (FC) of >2.0 or <0. 5, *q <*0.05, and a VIP value of >1 were selected as screening conditions to obtain significantly differential metabolites. They were subjected to a cluster analysis and presented as a heat map. Receiver operating characteristic (ROC) analysis was used to assess the use of metabolites as biomarkers for RH, and the area under the curve (AUC) was calculated using Youden index maximums’ analysis based on multivariate logistic regression model was performed to evaluate the value of the caffeine and its main metabolites combined as biomarker.

### Pathway Analysis

The MetaboAnalyst 4.0 software was used to analyze the biological pathways of differential metabolites, statistical tests were used to calculate the significance of the enrichment in each pathway. By referring to related literature, we made a biological explanation of the differences between the two groups.

## Results

### Baseline Characteristics

In total, 553 patients with T2DM were screened (65.6% male; mean age: 60.9 ± 8.5 years; diabetes duration: 8.28 ± 6.34 years; and HbA1c: 7.38 ± 1.6%). Of the 40 patients with RH, 8 (20%) had hypoglycemia ≥10 times within 6 months prior to the study. The characteristics of the participants are summarized in **Table 1**. The two groups were well matched after PSM, and no differences were found between the groups in terms of sex, age, HbA1c, duration of diabetes, blood pressure, BMI, TC, LDL-c, HDL-c, uric acid, eGFR, and treatment with glucose-lowering agents. The diet questionnaire showed that the number of regular tea or coffee users between the two groups was not statistically different. The triglyceride level in the DH group was lower than that of the DC group (1.90 ± 0.95 vs 1.28 ± 0.59, *P* = 0.003). Further details are shown in **Table 1**.

**Table 1:** Demographic and clinical characteristics.

Index	Before PSM			After PSM		
	T2DM reported noHypoglycemia(N=426)	T2DM reported hypoglycemia (N=127)	*P* value	T2DM control group (DC group)(N=40)	T2DM with hypoglycemia group (DH group)(N=40)	*P* value
Age (years)	60.95±8.58	60.60±8.30	0.681	61.95±7.20	60.78±8.29	0.530
Gender (male/female)	180/246	39/88	0.020*	14/26	18/22	0.503
BMI (kg/m^2^)	25.55±4.30	24.96±2.90	0.141	24.96±3.58	24.68±2.97	0.655
T2DM duration (years)	7.66±5.95	10.42±7.10	<0.001*	9.67±7.32	11.85±6.62	0.089
HbA1c (%)	7.42±1.71	7.25±1.31	0.228	7.22±1.68	7.40±1.38	0.562
FBG (mmol/L)	8.08±3.02	7.99±2.45	0.775	8.29±2.91	8.26±2.20	0.819
C-peptide (ng/mL)	2.55±1.16	2.30±1.17	0.039*	2.33±1.05	2.37±1.31	0.888
eGFR (ml/min/1.73m^2^)	97.11±15.20	97.79±14.49	0.569	96.56±11.53	98.18±10.74	0.439
TG (mmol/L)	1.82±1.63	1.57±0.88	0.106	1.90±0.95	1.28±0.59	0.003*
CHOL (mmol/L)	5.59±1.31	5.44±1.29	0.252	5.24±1.32	5.20±1.25	0.804
LDL (mmol/L)	3.09±0.90	2.99±0.94	0.277	2.95±0.97	2.84±0.94	0.581
HDL (mmol/L)	1.54±0.39	1.52±0.31	0.439	1.40±0.27	1.56±0.38	0.073
UA (mmol/L)	361.73±95.88	351.20±85.18	0.266	360.63±92.96	349.38±84.38	0.609
Hypertension, N (%)	230(54.0%)	78(61.4%)	0.139	28(70%)	26(65%)	0.815
consumption of tea or coffee, N (%)						0.372
< 1 time/week	–	–	–	23(57.5%)	27(70%)	–
1-6 times/week	–	–	–	5(12.5%)	4(10%)	–
≥ 1 time/day	–	–	–	12(30.0%)	9(20%)	–
Diabetes medications, N (%)						
Metformin	230(54.0%)	74(58.3%)	0.395	26(65%)	24(60%)	0.824
Sulfonylureas	151(35.4%)	62(48.8%)	0.007*	14(35%)	18(45%)	0.503
Glinides	51(12.0%)	13(10.2%)	0.299	6(15%)	7(17.5%)	0.964
Insulin	37(8.7%)	37(29.1%)	<0.001*	15(37.5%)	19(47.5%)	0.366

Data are presented as n (%), mean ± standard deviation, or median (interquartile range). * P-value <0.05. PSM: propensity score matching.

### Metabolomic Characteristics

**Figure 2:**
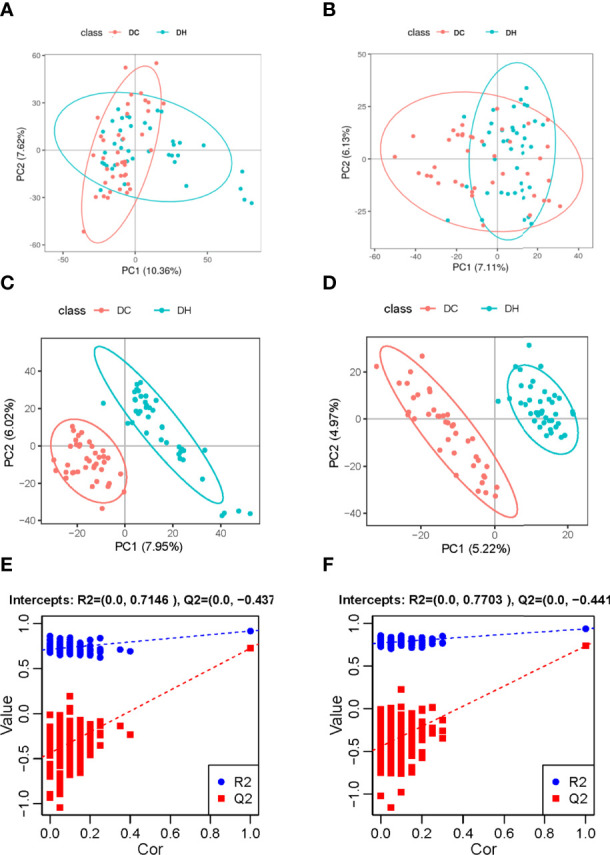
Multivariate analyses of serum metabolites. Score plot of the PCA model in the **(A)** positive and **(B)** negative ion modes. Score plot of the PLS-DA model in the **(C)** positive and **(D)** negative ion modes. PLS-DA model was conducted to identify the differences between the two groups. Distinct separations between the DC (red dots) and DH (blue dots) groups were presented. The PLS-DA models were validated using random permutation tests. The R2Y and Q2 intercepts were 0.61 and −0.44 in the positive mode **(E)** and 0.77 and −0.44 in the negative mode **(F)**, demonstrating that there was no overfitting and that the PLS-DA model was reliable. PCA, Principal component analysis; PLS-DA, projections to latent structures-discriminant analysis; T2DM, type 2 diabetes mellitus; DC, T2DM control group; DH, T2DM hypoglycemia group.

We evaluated the effect of RH exposure on serum metabolism using nontargeted, global metabolite profiling. According to XCMS records, 5884 metabolite features were detected in the positive mode, and 4674 were detected in the negative mode. From the features, a total of 836 metabolites were captured (374 in positive mode and 517 in negative mode) by preliminary screening. PCA was performed to obtain a comprehensive view (**Figures 2A**, **B**). PLS-DA, which has better classification and discrimination capacity, focuses on grouping and identifying the differences between the grouped samples. The PLS-DA model showed pronounced separations between the DC (red dots) and DH (blue dots) groups (**Figures 2C**, **D**), indicating significant metabolic differences between two groups. The PLS-DA models were validated using random permutation tests. R2 and Q2 were used to evaluate the quality of the model (**Figures 2E**, **F**, respectively). The results showed that there was no overfitting and that the PLS-DA model was reliable. The difference between each group was compared, and the FC was calculated.

### Differential Metabolites

**Figure 3:**
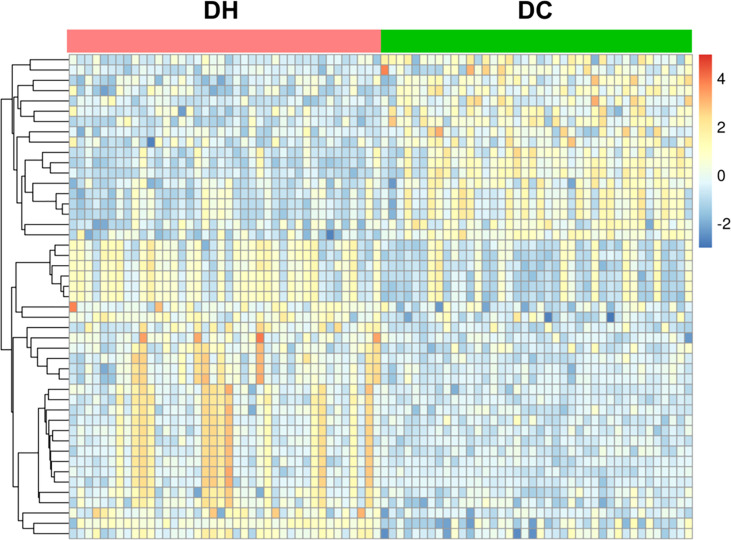
Heat map of cluster analysis of the differential metabolites. The abscissa represents DH group(red) and DC group(green) samples, the ordinate represents 47 differential metabolites. The color in the panel indicates the relative content of each metabolite: blue represents low levels, and yellow represents high levels.Cluster analysis showed that the differential metabolites were clearly grouped with high repeatability. T2DM, type 2 diabetes mellitus; DC, T2DM control group; DH, T2DM hypoglycemia group.

**Figure 4:**
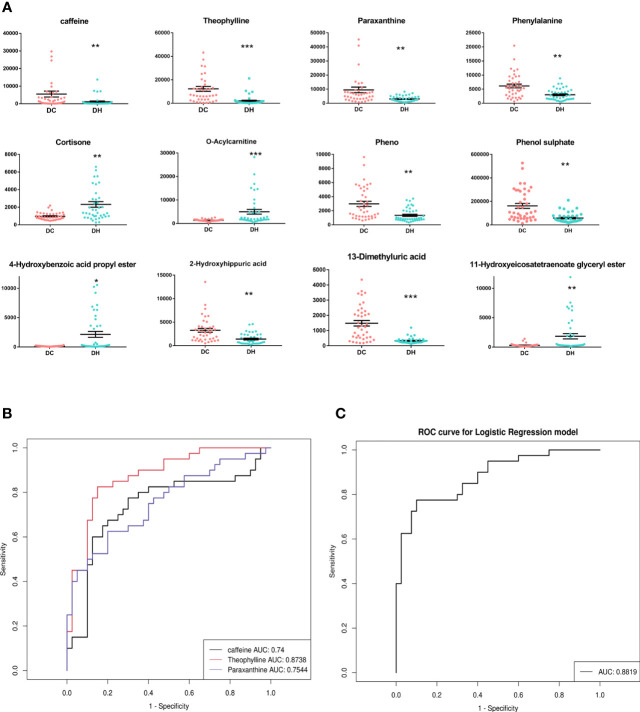
The significantly differential metabolites. **(A)** The relative content of 12 differential metabolites in two groups.*P < 0.05, ** *P* < 0.01, *** *P* < 0.001, DH vs. DC. **(B)** A ROC curves analysis was performed to evaluate the use of caffeine and its main metabolites as biomarkers for RH. Black represents caffeine, red represents theophylline, blue represents paraxanthine. **(C)** ROC curve for combinations of caffeine and its main metabolites(paraxanthine and theophylline). T2DM, type 2 diabetes mellitus; DC, T2DM control group; DH, T2DM hypoglycemia group; ROC, Receiver operating characteristic; AUC, area under the curve.

Based on the selection criteria (FC >2.0 or <0. 5, VIP >1, and *q <*0.05), 47 significantly differential metabolites were identified after HMDB database annotated. Cluster analysis of the differential metabolites showed that the metabolites were clearly grouped with high repeatability (**Figure 3**). These metabolites primarily included lipids and lipid-like molecules (23.81%), organic acids and derivatives (23.81%), organo heterocyclic compounds (23.81%), and benzenoids (23.81%). Among them, we identified 12 differential metabolites as potential markers to explain the variation between the DH and DC groups, as presented in **Table 2**. The DH group had higher levels of cortisone, O-Acylcarnitine, 11-hydroxyeicosatetraenoate glyceryl ester, and 4-hydroxybenzoic acid propyl ester and lower levels of caffeine, theophylline, paraxanthine, phenylalanine, phenol, phenol sulfate, 1, 3-dimethyluric acid, and 2-hydroxyhippuric acid than the DC group (**Figure 4A**).

**Table 2:** Significantly altered metabolites between DC and DH group by metabolomics analysis.

Metabolite	VIP	ROC	Fold change(DH/DC)	Trend	*q*-value
Caffeine Theophylline Paraxanthine Phe Cortisone O-Acylcarnitine Phenol Phenol sulphate 4-Hydroxybenzoic acid propyl ester 2-Hydroxyhippuric acid 1,3-Dimethyluric acid 11-Hydroxyeicosatetraenoate glyceryl ester	4.089 6.001 3.485 3.020 2.618 3.043 3.213 3.704 4.363 3.708 5.243 3.431	0.74 0.87 0.75 0.75 0.74 0.79 0.76 0.79 0.71 0.78 0.85 0.73	0.207 0.164 0.321 0.493 2.419 3.674 0.453 0.359 15.031 0.431 0.222 5.974	down down down down up up down down up down down up	0.005 <0.001 0.003 0.003 0.005 <0.001 0.002 0.001 0.015 0.001 <0.001 0.009

DC, type 2 diabetes mellitus control group; DH, type 2 diabetes mellitus with hypoglycemia group. ROC, Receiver Operating Characteristics.

### Caffeine Metabolites

Caffeine can be demethylated into paraxanthine (84%), theophylline (12%), and theobromine (4%) by cytochrome P450 1A2, a liver enzyme (26). In our study, the levels of caffeine and the main metabolites (theophylline and paraxanthine) were significantly lower in T2DM patients with RH. To explore the discriminative value of caffeine and the main metabolites, we performed a ROC curve analysis (**Figures 4B**, **C**). The AUCs of caffeine, theophylline and paraxanthine were 0.74, 0.87 and 0.75 respectively. The AUC improved (0.88) when the caffeine and two metabolites were combinated, show that caffeine and the main metabolites could be potential biomarkers for RH. We also employed serum molar concentration ratios of caffeine and paraxanthine to evaluate the activity of CYP1A2 (27) and found no difference between the groups (19.21 ± 34.74 vs 16.28 ± 23.17, *P* = 0.659).

### Metabolic Pathway Analysis

**Figure 5:**
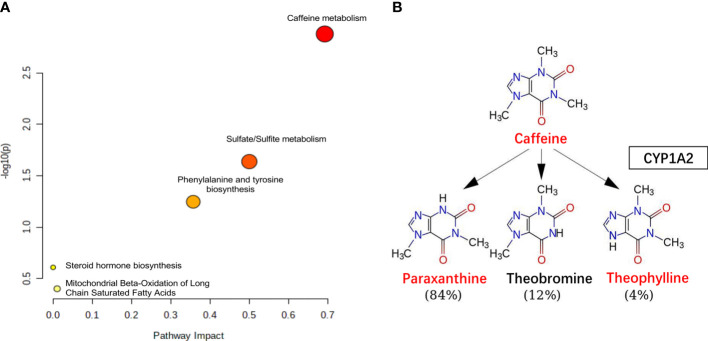
The pathway analysis. **(A)** Pathway analysis for significantly altered metabolites among the DC and DH groups. Pathway impact values are plotted against X-axis and -log (P) are plotted against Y-axis. For visual clarification, the pathway importance and the statistical significance are proportional to node radius and color, respectively. **(B)** Caffeine metabolism. Caffeine is demethylation *via* CYP1A2 in liver, the metabolites include paraxanthine, theophylline and theobromine. The levels of caffeine, theophylline, paraxanthine were lower (red color) in DH group than in the DC group. T2DM, type 2 diabetes mellitus; DC, T2DM control group; DH, T2DM hypoglycemia group.

Furthermore, we performed a pathway enrichment analysis of the 12 significantly different metabolites using the MetaboAnalyst software and the KEGG database to explore the metabolic pathways involved in RH onset or progression. They were enriched in the following five pathways (scatter plot in **Figure 5A**): caffeine metabolism (**Figure 5B**); sulfate/sulfite metabolism; phenylalanine and tyrosine biosynthesis; mitochondrial beta-oxidation of long-chain saturated fatty acids; and steroid hormone biosynthesis.

## Discussion

Recurrent episodes of hypoglycemia blunts counter-regulatory responses and leads to IAH, the detailed mechanism has not yet been elucidated. To date, very few studies have focused on the metabolism alteration during hypoglycemia. In the present study, we conducted a PSM matched case-control study to explore the serum differential metabolites using UPLC-MS, identified 12 potential biomarkers and 5 metabolic pathways strongly associated with the occurrence of RH. To the best of our knowledge, this is the first study to explore the metabolic responses to RH in T2DM.This work may help in the identification of clinical predictors biomarkers or potential therapeutic targets for RH.

### Caffeine Metabolism

Caffeine, the most widely consumed psychoactive agent (1,3,7-trimethylxanthine), is the basic component of coffee and tea and is also added in non-alcoholic energy drinks (28). Increasing evidence display the close connection between caffeine and many chronic diseases including T2DM, cardiovascular, neurological and liver diseases (29). Caffeine and its metabolites were identified as promising diagnostic biomarkers for some disease (30). Interestingly, we found that the serum concentrations of caffeine and its downstream metabolites were significantly lower in RH. Caffeine metabolite profiles may be reliable candidate biomarkers for RH. Future study can enlarge the sample size to make the predictive model more robust.

To further explore the possible relationship between caffeine metabolism and diabetic recurrent hypoglycemia, we reviewed the relevant literature. Recently, a large cohort study shows that the caffeine related metabolites had inverse associations with diabetes risk (31), provides new evidence that caffeine prevents T2DM. Furthermore, the benefits of caffeine for hypoglycemia management is attracting growing attention. Previous studies showed that modest amounts of caffeine or theophylline were associated with augmented counter-regulatory responses to insulin-induced hypoglycemia (32–35). Recent studies that arrived at similar conclusions that prior caffeine ingestion might be beneficial in reducing nocturnal (36) and exercise-associated hypoglycemia (37) risk in T1DM. The possible mechanisms include the following: 1) Under hypoglycemic conditions, an increase in cerebral blood flow would compensate for the decrease in glucose delivery, resulting in neuroglycopenia and the release of counter-regulatory hormones. Caffeine may uncouple the brain blood flow and glucose utilization *via* antagonism of adenosine receptors, simultaneously attenuating brain blood flow while augmenting brain glucose demand, leading to earlier response to glucose recovery (38). 2) Non-rapid eye movement (NREM) sleep has been implicated in suppressing the counter-regulatory response to hypoglycemia. Caffeine can reduce NREM sleep time, regulate physiological sleep (39), and attenuate the inhibition of the response to hypoglycemia. 3) Metabolites of caffeine may inhibit cyclic adenosine monophosphate (c-AMP) decomposition. The increased plasma c-AMP level mediates hepatic gluconeogenesis (40). However, existing studies on the improvement of caffeine to hypoglycemia are limited to T1DM and healthy populations in laboratory conditions, it remains unknown whether caffeine link to RH in clinical practice of T2DM. The present study showed the changes of caffeine metabolites in RH, might provide novel clinical evidence for caffeine as a potential treatment for hypoglycemia.

Caffeine is an exogenous compound that can only be obtained from food or medicine. Serum caffeine concentration is affected by caffeine intake, absorption, and the enzymes activity. Based on these considerations, we assessed caffeine intake and CYP1A2 activity, which displayed no difference. However, caffeine is present in a variety of food products. It is difficult to discern potential dietary sources of caffeine and to assess the intake level precisely in observational studies. Here we included the Chinese population living in Fujian where tea is more commonly consumed than coffee. Variation in the type of tea, cup size, and brew strength may result in the misestimation of caffeine intake. In this study, we used a UPLC-MS-based metabolomic analysis that reflects the most downstream metabolite information and effectively compensates for the disadvantage in intake assessment. This technique can help us define the potential association between caffeine metabolism and hypoglycemia, and further validation studies are needed.

### Lipid Metabolism

Exposure to hypoglycemia activates lipolysis and hydrolyzes triglycerides to produce glycerol and free fatty acids (FFAs) as substrates for the gluconeogenic process (41). Our study supported this view from a clinical perspective that has seldom been reported in previous studies. The conversion of FFAs to acyl-coenzyme A (CoA) is the first step in their degradation. Carnitines transport long-chain acyl-CoA into the mitochondria for β-oxidation to form acetyl CoA, which enters the tricarboxylic acid cycle (42). Carnitine is then converted into acylcarnitine, and excess acylcarnitine is released into the bloodstream. Thus, serum acylcarnitine reflects fatty acid metabolism (43). Our study showed a decrease in triglycerides and upregulation of O-Acylcarnitine in the DH group, indicating the acceleration of FFA β-oxidation and reflecting the adaptive changes of RH in T2DM.

### Other Changes

Our results demonstrated other metabolomic alterations with RH. Cortisol release in hypoglycemia provides a more slow and sustained method to maintain blood glucose, activates hepatic gluconeogenesis, and increases lipolysis to provide an alternative energy source. Previous studies have demonstrated that increased levels of cortisol during hypoglycemic clamp generally returns to normal within 24 hours (14). Cortisol responses to RH are seldom investigated (44). We observed a higher level in cortisone following RH in T2DM. Due to the retrospective study design, we did not identify the exact time interval between sample collection and occurrence of hypoglycemia, which seems to be a cortisone response adaptation to RH.

Under limited glucose conditions, amino acids can also contribute to providing energy. Phenylalanine can be converted into tyrosine in the presence of phenylalanine hydroxylase and can be used for energy production (45). Thus, we observed a lower level in phenylalanine concentration in the hypoglycemic group. In addition, both phenylalanine and tyrosine are catecholamine precursors involved in dopamine synthesis (46). The change in phenylalanine in our study might also be a response to the overconsumption of dopamine in RH. Moreover, phenylalanine can be used as an index of cardiovascular disease (CVD) (47). Magnusson demonstrated that the diabetes-predictive amino acid score (derived from tyrosine, isoleucine, and phenylalanine) strongly predicts the risk of CVD in T2DM (48), which appears to be partially mediated by the propensity for atherosclerosis, suggesting that disturbed amino acid metabolism may be a potential link to hypoglycemia and CVD. The same abnormality of phenylalanine metabolism was observed under hypoglycemic conditions in our study, which confirms the findings of previous studies.

## Limitations

First, the sample size of the investigation was relatively small, and the prognosis information was limited. Second, although the definition of RH used in this study is common in real-world hypoglycemia studies, the results would be more convincing if new techniques, such as continuous blood glucose monitoring, were taken into consideration in the definition. Third, our study is observational and unable to establish causality between hypoglycemia and caffeine consumption in T2DM. Future prospective intervention studies are required to confirm the association.

## Conclusion

To the best of our knowledge, this is the first study to explore the serum metabolic characteristic profile to RH in T2DM. We found that RH was associated with lower caffeine metabolism activity, showed that caffeine metabolites may be reliable predictor biomarkers for RH. Our findings also bring new clinical evidence for the beneficial effects of caffeine on hypoglycemic counter-regulation in the previous studies, provide a new strategy for the management of RH. In addition, we demonstrated the metabolic changes in lipid metabolism, phenylalanine and cortisone under RH, indicating the acceleration of FFA β-oxidation, thereby contributing to a further understanding of pathological mechanisms in RH.

## Data Availability Statement

The raw data supporting the conclusions of this article will be made available by the authors, without undue reservation.

## Ethics Statement

The studies involving human participants were reviewed and approved by the Ethics Committee of Fujian Medical University Union Hospital. The patients/participants provided their written informed consent to participate in this study.

## Author Contributions

WLJ: study design, data analysis, and manuscript writing. KS, WLX, HL, LXH, and QL: investigation and data acquisition. ZZ, WK, LXY, and ZM: interpretation of data. LL: project administration. All authors read the final manuscript and approved the submission. All authors contributed to the article and approved the submitted version.

## Funding

This work is supported by Fujian Science and Technology Innovation Joint Fund Project, China. (2017Y9060), Shanghai Health and Medical Development Foundation (DMRFP_I_03), Fujian Science and Technology Innovation Joint Fund Project, China. (2019Y9062), Natural Science Foundation of Fujian Province (2020J011012).

## Conflict of Interests

The authors declare that the research was conducted in the absence of any commercial or financial relationships that could be construed as a potential conflict of interest.

## Publisher’s Note

All claims expressed in this article are solely those of the authors and do not necessarily represent those of their affiliated organizations, or those of the publisher, the editors and the reviewers. Any product that may be evaluated in this article, or claim that may be made by its manufacturer, is not guaranteed or endorsed by the publisher.

## Abbreviations

AUC, area under the curve; BMI, Body mass index; c-AMP, Cyclic adenosine monophosphate; CoA, Conversion of FFAs to acyl-coenzyme; CVD, Cardiovascular disease; DH, Type 2 diabetes mellitus patients with recurrent hypoglycemia; DC, Type 2 diabetes mellitus patients without hypoglycemia; eGFR, estimated glomerular filtration rate; FC, Fold change; FFA, Free fatty acids; HbA1c, Glycated hemoglobin; HDL-C, High-density lipoprotein cholesterol; HMDB, Human Metabolome Database; KEGG, Kyoto Encyclopedia of Genes and Genomes; LC-MS, Liquid Chromatography- Mass Spectrometry; LDL-C, Low-density lipoprotein cholesterol; M/Z, mass to charge; NREM, Non-rapid eye movement; PCA, Principal component analysis; PLS- DA, Projections to latent structures-discriminant analysis; PQN, Probabilistic Classification; PSM, Propensity score matching; QC-RSC, QC-robust spline Batch Correction; RH, Recurrent hypoglycemia; ROC, Receiver Operating Characteristics; T1DM, Type 1 diabetes mellitus; T2DM, Type 2 diabetes mellitus: UPLC-MS, Ultra-high Performance Liquid; Chromatography-Mass spectrometry; VIP, Variables important in the projection; XCMS, X C Mass Spectrometry.
